# Improving organisational systems for diabetes care in Australian Indigenous communities

**DOI:** 10.1186/1472-6963-7-67

**Published:** 2007-05-06

**Authors:** Ross Bailie, Damin Si, Michelle Dowden, Lynette O'Donoghue, Christine Connors, Gary Robinson, Joan Cunningham, Tarun Weeramanthri

**Affiliations:** 1 Menzies School of Health Research, Institute of Advanced Studies, Charles Darwin University, Darwin, NT, Australia; 2 Northern Territory Department of Health and Community Services, Darwin, NT, Australia; 3 School for Social and Policy Research, Institute of Advanced Studies, Charles Darwin University, Darwin, NT, Australia

## Abstract

**Background:**

Indigenous Australians experience disproportionately high prevalence of, and morbidity and mortality from diabetes. There is an urgent need to understand how Indigenous primary care systems are organised to deliver diabetes services to those most in need, to monitor the quality of diabetes care received by Indigenous people, and to improve systems for better diabetes care.

**Methods:**

The intervention featured two annual cycles of assessment, feedback workshops, action planning, and implementation of system changes in 12 Indigenous community health centres. Assessment included a structured review of health service systems and audit of clinical records. Main process of care measures included adherence to guideline-scheduled services and medication adjustment. Main patient outcome measures were HbA1c, blood pressure and total cholesterol levels.

**Results:**

There was good engagement of health centre staff, with significant improvements in system development over the study period. Adherence to guideline-scheduled processes improved, including increases in 6 monthly testing of HbA1c from 41% to 74% (Risk ratio 1.93, 95% CI 1.71–2.10), 3 monthly checking of blood pressure from 63% to 76% (1.27, 1.13–1.37), annual testing of total cholesterol from 56% to 74% (1.36, 1.20–1.49), biennial eye checking by a ophthalmologist from 34% to 54% (1.68, 1.39–1.95), and 3 monthly feet checking from 20% to 58% (3.01, 2.52–3.47). Medication adjustment rates following identification of elevated HbA1c and blood pressure were low, increasing from 10% to 24%, and from 13% to 21% respectively at year 1 audit. However, improvements in medication adjustment were not maintained at the year 2 follow-up. Mean HbA1c value improved from 9.3 to 8.9% (mean difference -0.4%, 95% CI -0.7;-0.1), but there was no improvement in blood pressure or cholesterol control.

**Conclusion:**

This quality improvement (QI) intervention has proved to be highly acceptable in the Indigenous Australian primary care setting and has been associated with significant improvements in systems and processes of care and some intermediate outcomes. However, improvements appear to be limited by inadequate attention to abnormal clinical findings and medication management. Greater improvement in intermediate outcomes may be achieved by specifically addressing system barriers to therapy intensification through more effective engagement of medical staff in QI activities and/or greater use of nurse-practitioners.

## Background

Indigenous Australians experience disproportionately high morbidity and mortality due to diabetes, substantially more than can be explained simply by underlying high diabetes prevalence among these populations. For example, while the estimated prevalence of diabetes among Indigenous adults (10% to 30%) was 2–4 times higher than that of non-Indigenous Australians [1], hospital separation rates for diabetes were 10–15 times higher [2], and the death rate associated with diabetes for 35–54 year old Indigenous people was 27–35 times higher [3] when compared with non-Indigenous Australians. These national statistics indicate that there is a need to improve management of diabetes among Indigenous people at a primary care system level to prevent or delay diabetes-related complications.

In Australia, General Practice is the backbone of the mainstream primary care system, and is supported by funding from a national health insurance scheme (Medicare). Most primary care is provided by self-employed general practitioners (GPs), and services are reimbursed on a fee-for-service basis [4]. GPs in primary care act as 'gatekeepers', with access to specialist medical services being available only on their referral.

Primary care systems for Indigenous people are more complex, with three main service provider sectors: the Indigenous community controlled health service sector, State and Territory government funded and/or operated services, and general practitioners in private practice [5]. This complexity of Indigenous primary care systems is particularly evident in the Northern Territory (NT). About 90% of Indigenous people in the NT live in 'discrete Indigenous communities' [6], and a majority of these people access primary health care through community health centres, operating under a variety of funding and governance models, rather than private general practice.

Many Indigenous community health centres are overwhelmed by providing 'sickness care' for people who are acutely unwell. Clinical consultations in Indigenous community health centres, compared with those in mainstream general practice, tend to be more complex, with more new patients and more problems managed [7]. These high demands for acute care services are a result of high rates of illness in Indigenous communities [8]. Other major challenges include inadequate staff numbers and high staff turnover [8], geographic isolation, lack of resident (and infrequent visits by) general and specialist medical staff [9] and inadequate funding [5].

These challenges add to the internationally recognised inadequacy of acute care oriented systems for meeting the needs of patients experiencing chronic problems [10]. Regardless of whether the problem is heart disease, diabetes, or hypertension, effective management of chronic disease requires scheduled and regular patient visits to clinics for monitoring disease control, detecting complications, adjusting medications, and negotiating lifestyle changes. The criticism that follow-up of chronically ill patients tends to be sporadic, prevention underutilised, and the patient's role in disease management overlooked in many health systems [11] is generally applicable to Australian Indigenous primary care.

While acute care services will always be necessary, Indigenous community health centres must at the same time expand their systems to provide effective care for people with chronic illness. In 1997, the health authorities in the NT developed an integrated life course strategy to address chronic disease (the Preventable Chronic Disease Strategy), with a focus on maternal and child health, underlying determinants of health, lifestyle modification, and improved evidence based clinical practice for prevention, early detection and management of chronic disease[12]. A related development was the implementation in 1998 of trials of "coordinated care" [13], which aimed to examine the feasibility and impact of enhanced community control of funding and management of health services, and the introduction of best practice clinical guidelines and computerised information systems within two geographically defined regions in the NT. A more widely implemented initiative over recent years has aimed to improve paper-based recall systems in community health centres [14]. However, little is known about the general nature and use of systems in community health centres.

Against this background we developed a project to assess the impact of a quality improvement (QI) intervention on primary care systems, processes and intermediate outcomes of care for the management of diabetes in the remote Indigenous community primary care setting. This paper describes the experience and outcomes of two cycles of the QI intervention in 12 Aboriginal community health centres in the Top End of the NT, with a focus on understanding system related factors which hinder or facilitate improvements in outcomes of care.

## Methods

### Study location and selection of health centres

**Figure 1:**
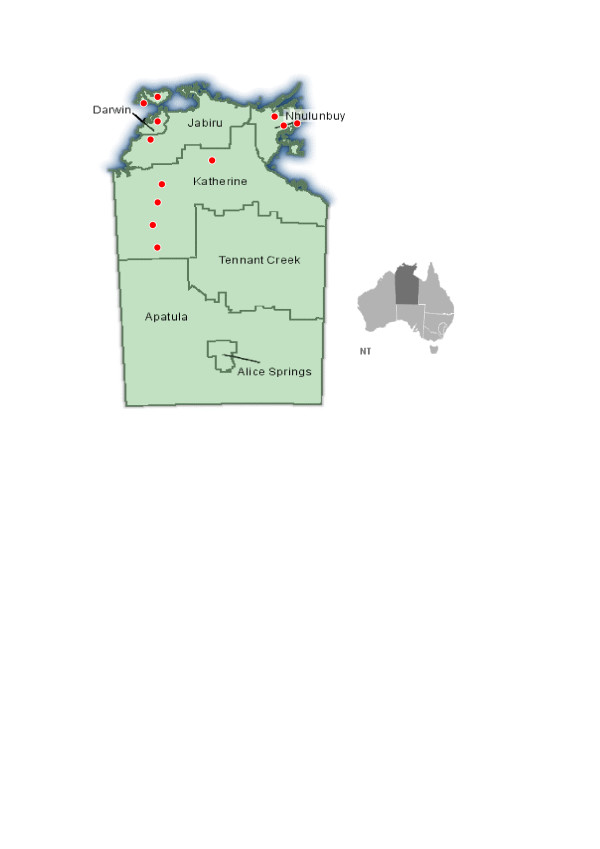
Distribution of 12 participating community health centres in the Top End of the Northern Territory, Australia.

This study was conducted in the Top End of the NT, an area occupying 522,561 square kilometres with an estimated resident population of 153,687 in 2003 [15]. Indigenous people comprise approximately 30% of the total population of the NT [15]. We purposively selected 12 health centres (Figure 1) from a total of 53 centres in the Top End, to reflect the diversity (rather than to be proportionally representative) of health centres in the region in terms of governance models (Indigenous community controlled, NT department of health funded/operated, and Health Board managed), population sizes (<500 residents, 500–999, and ≥1000), and remoteness (indicated by distance to the regional urban centres). Compared to all health centres in the Top End, health centres funded/operated by the NT department of health were under-represented in the sample, and health centres with medium-sized populations (500–999) were over represented. However, the 12 centres had good representativeness on remoteness distribution.

### The quality improvement (QI) intervention

Our QI intervention was conducted during 2002–2005 and featured two annual cycles of assessment, feedback, action planning and implementation:

#### Assessment

There were two distinct levels of assessment: first of health centre systems using an adapted version of the Assessment of Chronic Illness Care (ACIC) scale [16-18];. and second of quality of diabetes care via a patient record audit.

1) Our modified version of the ACIC scale included relatively detailed prompts relating to 34 items which were grouped into 7 components: organisational influence (3 items), external linkages (4 items), self-management (4 items), clinical decision support (3 items), delivery system design (9 items), clinical information systems (6 items), and integration (5 items). Compared with the original ACIC scale, this adapted version included three additional items (cultural competence, pathology management, and pharmacy management) in the delivery system design component, to reflect specific features of interest in NT centres. Staff (managers, doctors, nurses, Aboriginal health workers) were encouraged to discuss their perceptions using the prompts, and to score through consensus the level of development on a 0 (not at all) to 11 (fully developed) point scale. The mean was calculated from individual item scores to create a component score, and the mean of the 7 component scores formed the overall system score for the community health centre. The scale served both as a measurement tool and an intervention tool, as the discussion of system components led to a better understanding among staff of the quality of systems and consideration of how systems could be improved.

2) Quality of diabetes care was assessed through audit of a sample of clinical records in terms of processes of care (including guideline-scheduled services, medical treatments and medication adjustment) and intermediate patient outcomes (HbA1c, blood pressure, total cholesterol and ACR [Albumin Creatinine Ratio]). Our standardised audit form was based on previous research work in this area [19] and included a list of 27 scheduled service processes which local clinical guidelines recommend at specified intervals for people with diabetes [20]. A service was assessed as delivered if there was a record of delivery within the appropriate period preceding the audit.

We recorded prescribed medications for patients at the time of the audit. Dose information was collected for all hypoglycaemic, antihypertensive (including ACE inhibitor), lipid-lowering, and anti-platelet medicines.

We also assessed whether the adjustment of medication prescription was made among patients with HbA1c >8.0% and >7%, and with BP > 140/90 and 130/80 mmHg at any stage over the 12 months prior to the audit. While the clinical guidelines for the study population at the time of commencement of our study recommended treatment goals of ≤7% for HbA1c and ≤130/80 for blood pressure for individuals [20], we specified the two cut points for both HbA1c control and BP control to be used as indicators of quality of control. The medical regimes were considered as adjusted if the dose of a medicine was increased, an additional agent added, or a medicine substituted by another one.

Intermediate outcomes of diabetes care include four measures: all recorded values of HbA1c, blood pressure, total cholesterol and ACR in the 12 months prior to the audit. Patients were classified as hypertensive if there was a diagnosis of hypertension documented in medical records or they had blood pressure values >140/90 mmHg in three separate checks. Albuminuria was defined as having a diagnosis of renal disease documented in medical records or ACR > 3.4. Patients were classified as having hyperlipidemia if there was a diagnosis of hyperlipidemia documented in medical records or they had a total cholesterol level >5.5 mmol/L.

##### Audit sample

Community members who met all of the following criteria were included in the study population: 1) a definite diagnosis of type 2 diabetes according to health centre records; 2) identified as Aboriginal; 3) aged 16 years or older; and 4) lived in the community for 6 months or more during the previous 12 months. In the four community health centres where more than 30 eligible people were identified in each centre (the actual numbers were 42, 60, 76 and 155 respectively) a random sample of 30 records was drawn for audit. The randomisation was conducted by numbering the list of eligible patients and using a computer generated list of 30 random numbers to be included in the sample. In the eight other centres there were fewer than 30 eligible people identified in each, and records of all eligible people were audited. Overall, the records of 295 people with type 2 diabetes were included in the clinical record audit at baseline, which accounted for 57% of the eligible people with diabetes (N = 519). This sampling approach was designed to provide an adequate number of records to reflect the situation in each health centre rather than a representative sample of the population of people with diabetes.

**Figure 2:**
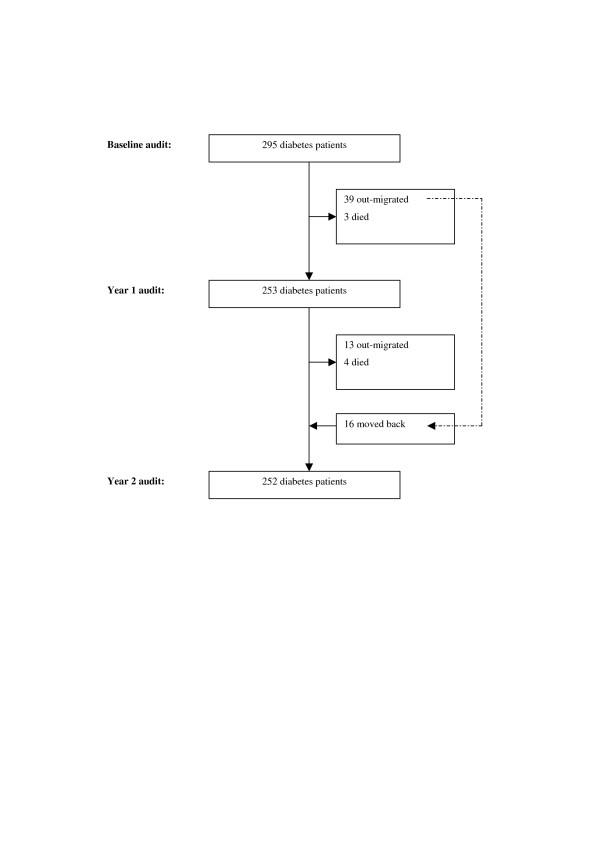
Follow-up of participants over the study period.

Of 295 participants at baseline, out-migration and death resulted in a loss-to-follow-up of 42 participants at the year 1 audit (n = 253) (Figure 2). With a further loss-to-follow-up of 17 people and moving back of 16 from previously out-migrated people, 252 participants remained for the year 2 audit.

##### Reliability of audit data

At each health centre, 10% of the records audited at baseline were selected randomly and audited again one to two months later by the same auditor for the same calendar period. Each health centre contributed 2 or 3 records, and the total reliability audit sample comprised 27 records. Intra-rater reliability for different types of audit items measured by Kappa statistics (κ), ranged from good to very good [21]: demographics (κ = 1.00), diabetes related complications (0.78–1.00), medications (0.91–1.00), and processes of care (0.74–1.00).

#### Orientation, feedback workshops and action planning

Early meetings with health centre staff and management were used as an opportunity to discuss the concepts of QI and the need to reorient services to address chronic illness effectively. As the project progressed we increasingly emphasised the value of identifying root causes of poor performance and the value of enhancing service systems rather than specific clinical services. Action planning workshops were held with staff and management of each health centre as part of each QI cycle to feed back systems assessment and clinical audit findings, compare performance with de-identified findings from other participating centres, to share lessons from the varied experience of health centres and to facilitate the development of action plans. Given their important and unique role, a learning conference was held to engage Aboriginal Health Workers and strengthen their understanding of the quality improvement intervention.

#### Implementation

Health centres carried out their action plans. Health centre staff were asked to document activities and initiatives relating to the implementation of action plans. The research team provided ongoing support to health centres in relation to implementation, mainly through email and telephone communications, and sometimes by site visits when necessary.

### Statistical analysis

Means and proportions were used to summarise normally distributed continuous and binomial data respectively, and 95% confidence intervals were calculated after adjustment for clustering by health centre. For data of non-Gaussian distribution, medians and interquartile ranges were used.

Changes in levels of health centre system development regarding chronic illness care (as represented by ACIC scores) and in quality of diabetes care (including processes and intermediate outcomes) over the study period were assessed by comparing the year 1 data (collected after one-year exposure to the intervention) and year 2 data (collected after 2-years-exposure), respectively, with the baseline data. As ACIC scores ranged from 0 to 11 and were measured at health centre level with a sample size of 12, it was difficult to determine whether the Gaussian assumption was met. Therefore, a non-parametric method, the Wilcoxon matched pairs signed rank sum test, was used for comparisons [21].

Multilevel regression models were used to assess changes in processes and intermediate outcomes of diabetes care. Three-level regression models were constructed to accommodate the inherent dependency structure of the data in the presence of both repeated observations from the same individuals (level 2 unit) and clusters within the same community health centre (level 3 unit) [22]. Two independent time variables, X_1 _(X_1 _= 0 denotes baseline period; X_1 _= 1 year 1) and X_2 _(X_2 _= 0 denotes baseline period; X_2 _= 1 year 2), were entered into the regression models separately, and their regression coefficients, respectively, indicated the average magnitude of changes in quality of care one year and two years after the intervention.

Two types of three-level regression models were employed according to the nature of dependent variables. First, three-level logistic regression models were used for dichotomous dependent variables, for example, process variable 'HbA1c was tested in previous 6 month (yes/no)' and intermediate outcome variable 'HbA1c level was less than 7.0% (yes/no)'. Regression coefficients were transformed into odds ratios using e^β^, and odds ratios then were converted into risk ratios by means of a published formula [23]. Second, three-level linear regression models were used for continuous dependent variables (eg values of systolic blood pressure). These analyses were conducted using Stata version 8.2 (Stata Corporation, College Station, Tex, USA). The command "gllamm" [generalised linear latent and mixed models] was used with the "family" option as "binomial" and the "link" option as "logit" for three-level logistic regressions. Three-level linear regressions were conducted using Stata command "gllamm" with the "family" option as "Gaussian" and the "link" option as "identity".

The study was approved by the Human Research Ethics Committee of the Northern Territory Department of Health & Community Services and the Menzies School of Health Research, and by its Indigenous health research sub-committee.

## Results

### Characteristics of the study participants at baseline

The records of 295 people with type 2 diabetes were audited at baseline (Table 1). The sample included fewer men than women (39% vs 61% at baseline). At baseline the median age of the participants was 48 years (range 16–87), with a high prevalence of smoking (36%) and microvascular complications (42%). Approximately one-half of the participants also had hypertension. Seventy-seven percent of participants had attended their community health centres within the previous 3 months.

**Table 1:** Characteristics of clinical record audit sample at baseline (N = 295)

Characteristic	Number (%), mean ± SD or median (interquartile range)
Median age (range), years	48 (16–87)
Men	116 (39%)
Median duration of diabetes*, years	5.7 (2.6, 8.7)
Current smokers	107 (36%)
	
Diagnosis of hypertension in medical record	140 (47%)
Diagnosis of hyperlipidaemia in medical record	84 (28%)
Any microvascular complications^†^	124 (42%)
Any macrovascular complications^‡^	57 (19%)
Medical comorbid conditions^§^	
0	86 (29%)
1	81 (27%)
2	63 (21%)
3	43 (15%)
4+	22 (8%)
	
Attended health centre in last 3 months	226 (77%)
Attended health centre in last 12 months	270 (92%)
Reasons for last attendance during prior 12 months	
Chronic illness care	164 (61%)
Acute care	88 (33%)
Sexual health or immunisation	18 (6%)

* Exclude 85 patients (29% of the total sample) whose date of diagnosis of diabetes was not documented in medical record.

† Microvascular complications include any retinopathy, nephropathy, neuropathy, renal disease, foot ulcer or amputation noted in the medical record.

‡ Macrovascular complications include any coronary artery disease or stroke noted in the medical record.

§ Medical comorbid conditions include hypertension, hyperlipidaemia, or any micro and macrovascular complications mentioned above.

### Change in health centre system development

Median ACIC component scores at the baseline ranged from 2.5 to 5.4 (Table 2). Scores improved progressively and significantly over the two year follow-up. During the course of the study health centre staff reported implementing a range of strategies and actions to improve health centre systems and services (Table 3). The strategies involved either increased resources or innovative activities that promoted and improved interaction between health care providers and patients. For example, most health centres included specific goals of chronic illness care in their business plans, with some lobbying their governing bodies to secure funds to support new positions for a nurse or chronic illness coordinator. The majority of strategies were carried out with the use of existing resources and by introducing creative initiatives to proactively engage community residents in chronic illness care. For example, a health centre, in conjunction with community organisations, ran community-based programs (i.e. 'No Drugs, No Violence, No Alcohol') to promote healthy lifestyle, and provided incentives to stimulate and reinforce behaviour changes. Changes in delivery system design were characterised by regular review of roles among primary care team members, designated staff time for home visits, and providing transport to clients for clinic visits.

**Table 2:** Changes in levels of system development over study period (Assessment of Chronic Illness Care scores, potential range 0–11)

**System components**	**Baseline**	**Year 1**	**Year 2**	Change (P value)* Year 1 – baseline	Change (P value)* Year 2 – baseline
			
	**Median (Interquartile Range)**				
Organisational influence	3.4 (2.5, 4.3)	5.2 (4.5,5.3)	6.1 (4.5, 6.7)	**+1.8 (0.002)**	**+2.7 (0.003)**
External linkages	4.8 (4.0, 5.6)	5.4 (4.6, 6.0)	6.5 (5.3, 7.8)	+0.6 (0.089)	**+1.7 (0.002)**
Self-management support	3.8 (3.0, 3.9)	5.3 (4.2. 5.8)	6.4 (4.9, 7.3)	**+1.5 (0.005)**	**+2.6 (0.004)**
Decision support	4.7 (4.0, 5.0)	5.8 (4.7, 6.3)	7.2 (5.8, 8.2)	+1.1 (0.077)	**+2.5 (0.025)**
Delivery system design	4.5 (3.6, 5.2)	5.9 (5.3, 6.7)	7.2 (5.8, 8.2)	**+1.4 (0.002)**	**+2.7 (0.007)**
Clinical Information systems	5.4 (4.6, 5.9)	5.9 (5.3, 7.3)	7.7 (6.1, 8.3)	**+0.5 (0.037)**	**+2.3 (0.015)**
Integration	2.5 (1.9, 3.5)	4.5 (3.5, 5.4)	5.1 (4.1, 6.5)	**+2.0 (0.002)**	**+2.6 (0.004)**
Overall (Total)	4.3 (3.4, 4.7)	5.6 (4.6, 6.0)	6.1 (5.6, 7.3)	**+1.3 (0.002)**	**+1.8 (0.002)**

* P values are for Wilcoxon matched pairs signed rank sum tests. Changes significant at 0.05 level are shown in bold.

**Table 3:** Examples of strategies and actions implemented by participating community health centres to improve services and systems

**Organisational influence**
▪ Dedicated new Medicare officer time to process Medicare claims
▪ Prepared for the Australian General Practice Accreditation Limited (AGPAL) accreditation
▪ Secured financial resources to fund a new nurse position
▪ Lobbied to recruit a program coordinator to coordinate chronic illness care
▪ Developed a business plan including specific chronic illness care goals
▪ Increased support from the regional Quality Improvement Coordinator to implement quality plans
▪ Increased efficiency in claiming for Medicare funded items, with funds then used to support operation and maintenance of the computerised information system

**External linkages**
▪ In conjunction with schools, Women's Centres, community stores or takeaways, ran chronic disease prevention programs
▪ Held regular meetings with new formed health advisory committee of Aboriginal elders, facilitating community input into health centre operation. The committee was also consulted to provide leadership and direction of community-based health activities
▪ Funded new community development positions with the health centre assisting with networking in the community and working on prevention
▪ Supported community-based initiatives, such as 'Water Aerobics'
▪ Assisted visiting nutritionist in organising the healthy lunch program in the community, and meal design and preparation for the Aged Care Centre
▪ Designated health centre time for community work on Friday am
▪ Organised an event titled "No Drug, No Violence, No Alcohol " in the community, and offered incentives, such as a return airfare to Darwin (donated by Qantas), to promote behaviour changes
▪ Ran a Diabetes Health Day in the community to improve clients' understanding of their conditions
▪ Supported the Nutrition Worker based at the community store to provide healthy foods
▪ Identified and established a list of outside services, names, and contact numbers to assist all staff to contact services and to help patients use them

**Self-management support**
▪ Designated chronic disease nurses to provide self-management support
▪ Implemented written care plans which contained patient goals agreed between clinicians and patients. These goals were reviewed during each visit
▪ Developed key local language concepts
▪ Arranged visiting mental health team (4 times/year) to assist with behaviour change interventions
▪ Helped setting up a Diabetes Action group in the community
▪ Designed oral guidelines and pictures to disseminate self-management knowledge and skills
▪ Enhanced smoking cessation services supported by pharmacy and availability of patches for nicotine replacement treatment
▪ Addressed concerns of patients and families through existing peer support groups at the Women's Centre in the community
▪ Provided diabetes patients with blood glucose self-monitoring materials
▪ Showed patients a food box (designed by a nutritionist) as an education tool to support individuals and families about what type of food is good for managing chronic disease
▪ Organised patient peer groups to share their stories about care, for example, buying a scrubbing brush to clean feet, rubbing feet with cream, wearing shoes, and buying good tucker from the store
▪ Supplied sharps containers for insulin dependent patients to safely dispose of needles
▪ Educated patients on general safe storage of medications
▪ Organised patient eduction delivered by the visiting Diabetes Educator from the Healthy Living NT

**Decision support**
▪ Provided training for primary care team by visiting specialists (eg physicians)
▪ Developed chronic disease flow sheet based on clinical guidelines (the CARPA)

**Delivery system design**
▪ Organised chronic disease days with presence of specialist providers
▪ Designated chronic disease nurses to implement planned visits and group visits, eg facilitating patients seeing multiple providers in a single visit
▪ Developed and implemented cross culture education and training programs for staff
▪ Held regular team meetings to revise and reinforce team roles
▪ Used registers to identify active and non-active participation of patients, analysed reasons for non-active participation, and took measures to improve
▪ When the doctor was visiting, Aboriginal health workers went out and talked to patients, and brought them into the health centre
▪ Put a sign up at the community shop for doctor's and physician's visits, and picked up people if necessary
▪ Arranged for patients to see specialists (eg ophthalmologists) in the regional centre, and transported them to their appointments
▪ Utilised interpreters provided by the Aboriginal Resource and Development Services
▪ Went out twice a day to give medications to patients in the community
▪ The health team met regularly every morning to do handover and assign responsibility for patient follow-up
▪ Commenced the use of case conferencing for patients with complex conditions and to assign responsibilities to PHC team members
▪ One afternoon a week was set aside to do home visits for patients with major chronic conditions, and documenting relevant information in the medical records
▪ Appointed the Health Centre Coordinator as team leader to ensure roles and responsibilities in chronic illness care

**Clinical information systems**
▪ Implemented a new electronic system (based on File Maker Pro) for recording of services, follow-up and reminding
▪ Performed a complete history notes audit and reorganised all individual files
▪ Installed a new computerised clinical information system, providing functions such as medical record-keeping, intelligent recalls, and featured appointments
▪ Developed and utilised a spreadsheet system with a list of people for follow-ups of 3 monthly bloods and the doctor's follow-ups of any abnormal findings
▪ Linked the health centre information system ("Communicare") with the regional information system "Health*Connect*" to realise transfer of information between settings and community health centres

### Change in quality of diabetes care

Delivery of guideline-scheduled processes of diabetes care increased progressively over the study period (Table 4). One year after the intervention, 9 out of a total of 27 service items showed statistically significant improvement, and this increased to 23 services after two years. Notably, finger prick or venous blood sugar level (BSL) test dropped from 61% to 51%, corresponding to an increasing use of HbA1c testing.

**Table 4:** Changes in processes of diabetes care

**Process items**	**Scheduled interval (months)**	**Baseline (n = 295)**	**Year 1 (n = 253)**	**Year 2 (n = 252)**	**Risk Ratios (95% CI)^† ^Year 1 vs baseline**	**Risk Ratios (95% CI)^† ^Year 2 vs baseline**
				
		**% of patients receiving services (95%CI)**				
**Basic measurement**						
Weight	3	47 (41, 53)	43 (37, 49)	61 (54, 67)	0.90 (0.72, 1.10)	**1.34 (1.14, 1.52)**
Height	Any time	32 (27, 38)	48 (42, 55)	71 (65, 76)	**1.61 (1.30, 1.91)**	**2.35 (2.08, 2.56)**
BMI	12	16 (12, 21)	21 (16, 26)	46 (40, 53)	1.24 (0.84, 1.76)	**3.09 (2.36, 3.82)**
Waist circumference	3	23 (18, 28)	28 (23, 34)	54 (48, 60)	1.28 (0.92, 1.70)	**2.42 (2.00, 2.82)**
BP	3	63 (57, 69)	63 (56, 68)	76 (71, 82)	0.94 (0.76, 1.10)	**1.27 (1.13, 1.37)**
**Eye check**						
Visual acuity	12	40 (35, 46)	42 (36, 48)	58 (52, 65)	1.06 (0.83, 1.31)	**1.49 (1.26, 1.71)**
Cataracts	12	28 (23, 34)	35 (29, 41)	24 (19, 30)	1.31 (0.97, 1.68)	0.80 (0.58, 1.08)
Fundi (dilated pupils)	12	34 (29, 40)	36 (30, 42)	30 (25, 37)	1.10 (0.83, 1.39)	0.90 (0.68, 1.15)
Ophthalmologist review	24	34 (29, 40)	41 (34, 47)	54 (48, 61)	1.28 (0.99, 1.57)	**1.68 (1.39, 1.95)**
**Feet check**						
Check done	3	20 (16, 25)	24 (19, 29)	58 (51, 64)	1.23 (0.84, 1.72)	**3.01 (2.52, 3.47)**
Sensation	3	9 (6, 13)	12 (8, 16)	48 (41, 54)	1.49 (0.87, 2.46)	**5.81 (4.43, 7.16)**
Peripheral pulses	3	8 (5, 12)	13 (9, 17)	48 (42, 54)	**1.79 (1.05, 2.92)**	**6.61 (5.00, 8.17)**
Pressure areas	3	7 (5, 11)	11 (7, 16)	44 (38, 51)	1.78 (0.99, 3.06)	**6.96 (5.10, 8.85)**
Infections	3	8 (6, 12)	12 (8, 17)	27 (22, 33)	1.64 (0.95, 2.69)	**3.56 (2.39, 5.03)**
**Laboratory investigations**						
BSL (finger prick or venous)	3	61 (55, 67)	51 (44, 57)	69 (63, 74)	**0.76 (0.60, 0.92)**	1.13 (0.97, 1.27)
HbA1c	6	41 (35, 47)	61 (55, 67)	74 (68, 80)	**1.54 (1.31, 1.75)**	**1.93 (1.71, 2.10)**
Total cholesterol	12	56 (50, 62)	70 (64, 76)	74 (68, 79)	**1.29 (1.13, 1.42)**	**1.36 (1.20, 1.49)**
Urine – Dipstix	3	20 (15, 25)	24 (19, 30)	48 (42, 55)	1.29 (0.93, 1.74)	**2.66 (2.15, 3.16)**
Creatinine	12	65 (59, 71)	68 (62, 74)	74 (69, 80)	1.03 (0.88, 1.16)	**1.15 (1.02, 1.26)**
ACR	12	54 (48, 59)	54 (47, 60)	61 (55, 67)	0.99 (0.80, 1.16)	1.15 (0.99, 1.31)
**Counselling/advice**						
Diet	3	15 (11, 19)	23 (18, 29)	36 (30, 42)	**1.73 (1.17, 2.45)**	**2.54 (1.90, 3.26)**
Activity	3	13 (9, 17)	22 (17, 27)	37 (31, 43)	**1.82 (1.21, 2.62)**	**2.97 (2.20, 3.83)**
Smoking	3	10 (7, 14)	21 (16, 26)	30 (25, 37)	**2.30 (1.50, 3.36)**	**3.21 (2.24, 4.37)**
Alcohol	3	9 (6, 13)	21 (16, 27)	33 (27, 39)	**2.59 (1.67, 3.82)**	**3.78 (2.64, 5.11)**
Diabetes medications	3	10 (7, 14)	26 (21, 32)	36 (30, 42)	**2.95 (1.94, 4.20)**	**3.65 (2.63, 4.82)**
**Immunisations**						
Influenza vaccination	12	54 (48, 59)	47 (41, 53)	83 (78, 87)	0.83 (0.65, 1.01)	**1.63 (1.50, 1.72)**
Pneumococcal vaccination	5 yrs	73 (68, 78)	72 (66, 77)	80 (74, 85)	0.91 (0.73, 1.06)	1.10 (0.99, 1.18)

**† **Calculated by using multilevel logistic regression models with adjustment for health centre clustering and repeated measurements within the same individuals, and by converting odds ratios into risk ratios using a published formula. Risk ratios significant at 0.05 level are shown in bold.

However, the proportion of patients prescribed insulin, oral hypoglycaemic agents, ACE inhibitors for those with albuminuria, aspirin for those with established CVD and treatment for hypertension and hyperlipidemia (statins) at year 2 was not significantly different to baseline (Table 5). There was an increase in prescription of aspirin for those with CVD risk factors at both years 1 and 2.

**Table 5:** Changes in pharmacological treatment rates over the study period

**Treatment**	**Baseline**	**Year 1**	**Year 2**	**Risk Ratios (95% CI)^† ^Year 1 vs baseline**	**Risk Ratios (95% CI)^† ^Year 2 vs baseline**
Any insulin use	10% (29/295)	11% (29/253)	10% (24/252)	1.17 (0.96, 1.40)	0.97 (0.79, 1.19)
Oral hypoglycaemic agents only	69% (203/295)	72% (181/253)	71% (180/252)	1.05 (0.83, 1.21)	1.08 (0.92, 1.20)
Hypertension on treatment	78% (112/143)	89% (111/125)	75% (119/159)	**1.16 (1.04, 1.22)**	0.96 (0.79, 1.09)
Albuminuria on ACE inhibitor	85% (129/151)	85% (124/146)	72% (114/158)	1.02 (0.86, 1.11)	0.87 (0.69, 1.00)
Hyperlipidemia on statin	71% (99/140)	83% (128/154)	67% (98/146)	**1.34 (1.18, 1.39)**	0.93 (0.75, 1.09)
Coronary heart disease/stroke on aspirin	56% (32/57)	60% (30/50)	70% (30/43)	1.00 (0.46, 1.47)	1.30 (0.85, 1.59)
Aspirin use among diabetes patients without cardiovascular events but with one or more other cardiovascular risk factors*	31% (53/173)	55% (91/165)	55% (101/184)	**2.90 (2.11, 3.15)**	**2.21 (1.67, 2.62)**

* Other cardiovascular risks refer to hypertension, hyperlipidemia and smoking.

† Risk ratios significant at 0.05 level are shown in bold.

Based on documentation in medical records, after identification of elevated HbA1c and BP, there was an increase in numbers of elevated results reviewed by a doctor, and consequently, an increase in medication adjustment rates at the year 1 audit (Table 6). However, these improvements were not maintained at the year 2 follow-up, with a significant drop in reviewing elevated results and medication adjustment during the year 2 period, even below the baseline level. Over the same intervals, the number of doctors employed (FTE) decreased from 9.5 at baseline to 9.3 at year 1 to 5.3 at year 2.

**Table 6:** Frequency of elevated HbA1c and blood pressure results followed by documentation of review by a doctor and medication change over the study period

**Follow up**	**Baseline**	**Year 1**	**Year 2**	**Risk Ratios (95% CI)^‡ ^Year 1 vs baseline**	**Risk Ratios (95% CI)^‡ ^Year 2 vs baseline**
HbA1c recorded in previous 12 months*	291	296	317		
Elevated HbA1c^† ^(%)	173 (59%)	179 (60%)	169 (53%)	1.02 (0.89, 1.16)	0.90 (0.78, 1.03)
Reviewed by a doctor (%)	45 (26%)	82 (46%)	4 (2%)	**1.76 (1.31, 2.37)**	**0.09 (0.03, 0.25)**
Medication adjusted (%)	18 (10%)	43 (24%)	5 (3%)	**2.31 (1.39, 3.84)**	**0.28 (0.11, 0.75)**
					
BP recorded in previous 12 months*	615	559	588		
Elevated BP^† ^(%)	145 (24%)	154 (28%)	151 (26%)	1.17 (0.96, 1.42)	1.09 (0.89, 1.33)
Reviewed by a doctor (%)	30 (21%)	62 (40%)	5 (3%)	**1.95 (1.34, 2.82)**	**0.16 (0.06, 0.40)**
Medication adjusted (%)	19 (13%)	33 (21%)	5 (3%)	1.64 (0.98, 2.74)	**0.25 (0.10, 0.66)**

* As multiple HbA1c and BP results might be documented in the medical record for each patient in previous 12 month period, we only collected, when available, the two most recent HbA1c results and the three most recent BP results for each audit interval.

† Elevated HbA1c was defined as HbA1c > 8.0%. Elevated BP was defined as BP > 140/90 mmHg.

‡ Risk ratios significant at 0.05 level are shown in bold.

The mean HbA1c improved significantly from 9.3% at the baseline to 8.9% at the year 1 audit, and maintained a mean of 8.9% at the year 2 audit (Table 7). The proportion of people with acceptable glycaemic control (HbA1c < 8.0%) and ideal glycaemic control (HbA1c < 7.0%) increased from 37% (baseline) to 46% (year 2), and from 19% to 28%, respectively. However, blood pressure control, total cholesterol control and maintenance of renal function (measured by ACR) largely remained unchanged over the study period.

**Table 7:** Changes in intermediate patient outcomes over the study period

**Intermediate outcomes**	**Baseline**	**Year 1**	**Year 2**	**Mean differences or Risk Ratios (95% CI)^† ^Year 1 vs baseline**	**Mean differences or Risk Ratios (95% CI)^† ^Year 2 vs baseline**
			
	**Mean or percentage (95% CI)**				
**HbA1c**					
Mean HbA1c level (%)	9.3 (8.8, 9.8)	8.9 (8.3, 9.4)	8.9 (8.6, 9.3)	**-0.4 (-0.7, -0.1)**	**-0.4 (-0.7, -0.1)**
HbA1c < 8%	37 (28, 46)	40 (30, 50)	46 (40, 52)	1.18 (0.79, 1.61)	**1.43 (1.03, 1.82)**
HbA1c < 7%	19 (13, 24)	21 (13, 29)	28 (22, 34)	1.16 (0.58, 2.09)	**1.74 (1.11, 2.50)**
**Blood pressure (mmHg)**					
Mean systolic BP	130 (127, 133)	131 (128, 135)	130 (126, 133)	2.3 (-0.6, 5.2)	-0.2 (-3.2, 2.8)
Mean diastolic BP	79 (77, 82)	79 (77, 82)	79 (76, 81)	0.4 (-1.6, 2.3)	-0.3 (-2.1, 1.4)
BP < 140/90	65 (58, 72)	59 (51, 67)	67 (61, 73)	0.84 (0.66, 1.02)	1.04 (0.90, 1.17)
BP < 130/80	33 (25, 41))	33 (23, 43)	29 (22, 36)	0.97 (0.69, 1.29)	0.80 (0.57, 1.10)
**Total cholesterol (mmol/L)**					
Mean total cholesterol level	4.9 (4.7, 5.1)	4.9 (4.6,5.2)	4.9 (4.6, 5.3)	-0.01 (-0.2, 0.2)	-0.06 (-0.3, 0.2)
Total cholesterol < 5.5	73 (66, 80)	73 (64, 82)	73 (63, 84)	1.00 (0.77, 1.17)	1.02 (0.82, 1.16)
Total cholesterol < 4.0	22 (16, 28)	24 (17, 31)	30 (19, 41)	1.22 (0.68, 1.97)	1.58 (0.95, 2.35)
**ACR**					
Median ACR level *	18.0 (3.7, 63.9)	19.6 (3.5, 83.2)	18.7 (4.8, 67.2)	P = 0.49	P = 0.32
ACR ≤ 3.4	21 (13, 29)	24 (13, 35)	18 (13, 23)	0.93 (0.42, 1.78)	0.76 (0.35, 1.46)
3.4 < ACR ≤ 34	41 (33, 49)	33 (24, 42)	43 (35, 51)	0.73 (0.43, 1.11)	1.05 (0.72, 1.40)
ACR > 34	38 (29, 47)	43 (30, 56)	39 (30, 48)	1.60 (0.92, 2.16)	1.13 (0.67, 1.63)

* Figures in this row are medians (interquartile ranges). P values indicate statistical significance based on Wilcoxon-Mann-Whitney test for non-parametric group comparison.

† Calculated by using multilevel regression models with adjustment for health centre clustering and repeated measurements within the same individuals, and by converting odds ratios into risk ratios when appropriate using a published formula. While the data for duration of diabetes were not sufficiently complete to allow for adjustment in the analysis (29% of participants had no date of diabetes diagnosis documented in medical records), additional adjustment for age did not significantly change the results. Therefore, we presented the data with no adjustment for age and duration of diabetes in this table. Mean differences or risk ratios significant at 0.05 level are shown in bold.

## Discussion

The study health centres initiated and implemented a number of system changes over the two completed QI cycles, and these developments are reflected in improvements in the ACIC scores for all components of health centre systems. Over the same period there was an improvement in quality of diabetes care as reflected in adherence to guideline-scheduled processes of care, but limited increases in attention to abnormal findings and medication adjustment. There was an improvement in HbA1c control, but not in BP control or cholesterol levels.

Our findings on systems improvement are very similar to those found in the first of the "Breakthrough" series in the US [24] despite their inclusion of hospitals and academic centres in addition to community based services. Compared with the baseline data of our study, the participating organisations had higher ACIC scores for organisational influence and self-management support, but similar scores for the other four components [18,25]. Similar improvements in processes of care and in HbA1c measures were also reported after one year of a QI intervention in 19 community health centres serving predominantly disadvantaged populations in the Midwest of the USA [26]. However, the intensity of the monthly cycles in the Midwest study was perceived by staff to be too burdensome, and potentially unsustainable. Our findings suggest quality improvement interventions featuring conventional, annual QI cycles can be comparably effective in achieving improvement in diabetes care and may be more likely to be institutionalised.

Improvements in key process measures, including HbA1c and BP monitoring, were generally greater than for other interventions in similar settings in Australia [19,27]. Although diabetes patients in our study experienced moderate improvement in HbA1c control, no significant improvement was found in other important intermediate outcomes, namely blood pressure and total cholesterol control. Difficulties in improving patient outcomes have been increasingly reported by studies focusing on diabetes quality improvement interventions [28-30]. For example, in a systematic review [28] assessing effectiveness of organisational and professional interventions on quality of diabetes care in primary care settings, Renders *et al *identified 13 studies that reported effects on both processes and outcomes of diabetes care. However, only 7 of these studies demonstrated a favourable effect on patient outcomes in addition to a positive effect on processes of care. In a recently published randomised controlled trial [30], O'Connor and colleagues reported implementation of a seven-step quality improvement change process in primary care medical practices. After the 2.5-year implementation period, the intervention failed to improve HbA1c, blood pressure, and LDL levels among patients, although most processes of care, including annual test rates of HbA1c, blood pressure and LDL, were significantly improved.

An increasing number of commentators have ascribed difficulties in improving patient outcomes to 'clinical inertia' (defined as failure of health care providers to initiate or intensify therapy when evidence-based treatment goals are not achieved) [31-35] but there has been a lack of longitudinal data to support this contention. Our data shows seriously inadequate medical review and medication adjustment following abnormal clinical findings and investigations (notably BP and HbA1c), with an inadequate improvement after feedback of these findings. For example, medication adjustment rates following identification of elevated HbA1c improved from 10% at baseline to 24% at year 1, but dropped to 3% at year 2. Over the same intervals, the number of doctors employed (FTE) dropped by 44%. It is likely that a certain and stable level of doctors working at health centres provides a basis for improvement in medication adjustment. Conversely, a serious shortage of doctors significantly impedes efforts for improvement. While there were relatively high rates of prescribing of ACE inhibitors, of aspirin and relatively good control of BP, the findings confirm that failure to intensify medical management is likely to be an important factor in the limited improvement in patient intermediate outcomes. This highlights the importance for medical staff to be effectively engaged in QI interventions and for the potential of nurse-practitioners to adjust medications according to protocols.

The experience of our quality improvement intervention suggests that increasing medication adjustment for diabetes patients in primary care systems can be challenging and complex. For example, while most computerised information systems used by participating heath centres provided prompts to health care providers that a test (e.g. HbA1c) was due, none of these systems could further classify that patient results were at evidence-based goals or not, and no prompts were provided to providers regarding how therapy could be intensified for patients to achieve their clinical goals. However, if future diabetes quality improvement interventions are expected to improve patient outcomes, medication adjustment measurements should be routinely included in the spectrum of quality of care measures, and barriers to making medical regimen changes in healthcare systems need to be carefully identified, and effective strategies for overcoming these barriers need to be tested and implemented.

While we aimed to include a cross section of services in terms of remoteness, population size and governance arrangements, the requirements of the participatory action process meant our selection of services was influenced to an extent by the likelihood of achieving active participation. The extent to which the findings are generalisable may therefore be limited. The high rate of adoption of QI processes by many services in the region (at least partly as a result of this study) has limited the value of conducting a comparative analysis with a non-intervention group. The lack of a comparison group in the analysis presented here and the two year duration of our time series study does not provide a high level of evidence regarding causal relationships between the QI intervention and process or outcome measures. While the reliability of our process and outcome measures obtained through the use of the audit tools has been shown to be good to very good, the facilitated self-report approach to use of the ACIC scale leaves some potential for bias in assessment of system development. However, this is expected to be less than for other projects which have not used a facilitator to standardise reporting and where the tool has fewer standardised prompts.

Encouraged by the experiences and impressive improvement achieved by participating health centres, we are currently extending the project to other jurisdictions to establish a national collaborative with a view to investigating how the quality improvement intervention developed in this study can be introduced and supported as routine practice in Australian Indigenous primary care settings. The collaborative will include service providers from a number of other states. In addition, the extension project will also test whether these quality improvement approaches can be applied to chronic conditions other than diabetes, including hypertension, coronary heart disease, renal disease, and mental illness. With a longer term follow-up over a larger number of health centres, it will provide better evidence on the nature of the causal relationship between QI interventions and quality of care.

## Conclusion

This QI intervention has proved to be highly acceptable in the Indigenous Australian primary care setting and has been associated with significant improvements in systems and processes of care and some intermediate outcomes. Improvements appear to be limited by clinical inertia with inadequate attention to abnormal clinical findings or the intensification of medication management. Greater improvement in intermediate outcomes may be achieved with more effective engagement of medical staff in QI activities and greater use of nurse-practitioners. Sustaining and continuing the improvements in care will require ongoing organisational commitment to support health centre staff to actively engage in ongoing QI cycles, with re-invigoration and refinement of successful strategies.

## Competing interests

The authors declare that they have no competing interests.

## Authors' contributions

R Bailie played the lead role in conceptualisation, design, management of fieldwork and drafting of this manuscript; D Si played a major role in reviewing the international literature, conceptualisation, data analysis and drafting of the manuscript; M Dowden and L O'Donoghue developed the participatory approach and conducted the fieldwork; C Connors and T Weeramanthri contributed to conceptualisation, design and facilitated engagement with health services; G Robinson and J Cunningham contributed to conceptualisation and design. All authors contributed to the interpretation of findings and revision of the manuscript.

## Pre-publication history

The pre-publication history for this paper can be accessed here:


